# Identification of Wheat Genotypes with High Tolerance to Combined Salt and Waterlogging Stresses Using Biochemical and Morpho-Physiological Insights at the Seedling Stage

**DOI:** 10.3390/plants14091268

**Published:** 2025-04-22

**Authors:** Saad Elhabashy, Shuo Zhang, Cheng-Wei Qiu, Shou-Heng Shi, Paul Holford, Feibo Wu

**Affiliations:** 1 Department of Agronomy, College of Agriculture and Biotechnology, Zhejiang University, Zijingang Campus, Hangzhou 310058, China; saad.elhabshy@alexu.edu.eg (S.E.); zmike@zju.edu.cn (S.Z.); qiucw@zju.edu.cn (C.-W.Q.); shouhengshi@zju.edu.cn (S.-H.S.); 2 Department of Crop Science, Faculty of Agriculture, Alexandria University, Alexandria 21545, Egypt; 3 School of Science, University of Western Sydney, Penrith, NSW 2751, Australia; p.holford@westernsydney.edu.au

**Keywords:** wheat, salinity, waterlogging, reactive oxygen species, antioxidative activities

## Abstract

Developing crop varieties with combined salinity and waterlogging tolerance is essential for sustainable agriculture and food security in regions affected by these stresses. This process requires an efficient method to rapidly and accurately assess the tolerance of multiple genotypes to these stresses. Our study examined the use of a pot trial in combination with the assessment of multiple traits to assess the tolerance of 100 wheat (*Triticum aestivum* L.) genotypes sourced from around the world to these combined stresses. The stresses were imposed on the plants using 100 mM NaCl and by submerging the root systems of the plants in their bathing solutions. The data gathered were subjected to principal component analysis (PCA), and an integrated score (IS) for each genotype was calculated based on multiple morpho-physiological traits; the score was used to rank the genotypes with respect to tolerance or susceptibility. There were significant differences among the 100 wheat genotypes in terms of the relative reductions in their growth parameters and chlorophyll contents, suggesting a rich, genetic diversity. To assess the accuracy of this methodology and to gain insight into the causes of tolerance or susceptibility, the five most tolerant (Misr4 (W85), Corack (W41), Kzyl-Sark (W94), Hofed (W57), BAW-1157 (W14)), and two least tolerant (Livingstong (W60) and Sunvale (W73)) genotypes were selected based on their IS and PCA analysis. These genotypes were then grown hydroponically with and without salinity stress. The data from this second trial were again subjected to PCA, and their IS were calculated; there was reasonable agreement in the ranking of the genotypes between the two trials. The most tolerant genotype (W85; Misr4 from Egypt) and most susceptible genotype (W73; Sunvale from Australia) were then examined in further detail in a third trial. Plants of Misr4 (W85) had lower Na^+^/K^+^ ratios, higher superoxide dismutase, peroxidase, catalase, and ascorbate peroxidase activities, and higher glutathione concentrations. As a result, plants of Misr4 (W85) had lower concentrations of reactive oxygen species (H_2_O_2_ and O_2_^•−^) and malondialdehyde than those of Sunvale (W73). This study offers an efficient methodology for the assessment of multiple sources of germplasm for stress tolerance. It has also identified germplasm that can be used for future breeding work and for further research on the mechanisms of tolerance and susceptibility to combined salinity and waterlogging stresses.

## 1. Introduction

The simultaneous occurrence of stresses due to salinity and waterlogging is more detrimental to crop growth and development than these stresses applied singly [1,2], and increased salinity and waterlogging in coastal areas is occurring due to rises in sea levels as a result of climate change [3]. Globally, salt-affected areas cover around one billion hectares, which is approximately 25–30% of all irrigated lands [4], and more than 77 million ha of land is at risk from soil salinization [5]. Waterlogging also affects a large portion of the world, negatively impacting approximately 10–12% of the global land area [6], and has been reported worldwide in regions such as Egypt and Saudi Arabia [7], in the mid-lower reaches of the Yangtze River in China [8], and in the Indo-Gangetic Plain in India [9]. Waterlogging is an issue, as it decreases the air available in the soil, resulting in hypoxia, which reduces plant growth [10]. Together, salinity and waterlogging are estimated to affect over 80 million ha [11]. These two stresses are often interrelated, as waterlogging can lead to land salinization by bringing salts to the surface [12]. In many regions of the world, such as Egypt, Australia, the USA, Pakistan, India, Iran, and Thailand, these two environmental stresses coexist [12]. Consequently, various plant species experience greater reductions in growth in saline-waterlogged environments compared with saline or waterlogged conditions alone [1,12,13]. While the physiological and molecular mechanisms of plant responses to individual environmental restrictions have been extensively studied, there is a paucity of research addressing the putative mechanisms that confer tolerance to combined stresses in plants [14].

Salinity stress can hinder the ability of plant root cells to absorb water from the soil and also induces oxidative stress due to the production of excess reactive oxygen species (ROS) [15], ion toxicities [16], water scarcity [17], nutritional imbalances, and alterations in metabolic processes that slow the rate of photosynthesis [18]. In addition, salinity causes the oxidation of proteins, alterations in DNA sequences [19], and strand breaks and crosslinks [20]. These stresses cause cell death, thereby impairing growth and plant development [21,22]. Reactive oxygen species consist of superoxide ions (O_2_^•−^), hydrogen peroxide (H_2_O_2_), hydroxyl radicals (OH^•^), and singlet oxygen (^1^O_2_) [7]. Plants alter their enzymatic and non-enzymatic antioxidant systems to fight oxidative stresses and have developed internal resistance mechanisms to mitigate the harmful effects of ROS and are highly redox-buffered due to water-soluble antioxidants such as glutathione [23] and ascorbate [24]. Salt-tolerant plants activate several enzymes to reduce ROS concentrations. Among these, superoxide dismutase (SOD) [25], peroxidase (POD) [24], catalase (CAT) [26], and ascorbate peroxidase (APX) [27] are the most important. Malondialdehyde (MDA) is widely recognized as a byproduct of membrane lipid peroxidation caused by ROS [28,29], and numerous studies have utilized MDA levels together with antioxidant enzyme activities as physiological indicators of stress tolerance [28,30,31]. In addition, saline-waterlogged environments disrupt energy-dependent ion discrimination at the root surface, leading to either reduced exclusion or increased uptake of Na^+^ [32], which affects Na^+^-to-K^+^ ratios; these ratios have also been used as indicators of stress tolerance.

Salt stress is responsible for about 60% of crop production losses [33]. Annually, an estimated 10 to 15 million ha of wheat cultivation is affected by heavy rainfall and consequent waterlogging worldwide that results in yield losses of around 20–50% [34]. Wheat (*Triticum aestivum* L.) is the most commonly cultivated cereal crop [35] but is well known for its sensitivity to salinity and waterlogging [36,37]. Providing salt- and waterlogging-tolerant varieties that are better adapted to regionally prevailing abiotic stresses is an excellent way to ensure successful crop growth to meet the grain production needs of the increasing human population [38]. Unfortunately, the complexity of these conditions and a lack of appropriate genetic resources have impeded the development of tolerant cultivars.

Finding the best genetic donors that can be used in the development of cultivars tolerant of combined salt and waterlogging stresses is the first and most important step in developing new cultivars, and many morphological and physiological traits have been assessed to identify tolerant genotypes [28]. Several studies have successfully evaluated the ability of wheat to withstand salt stress using multivariate analysis despite the fact that many agronomic traits have low heritability [39,40]. The use of an integrated score (IS) consisting of multiple combined traits together with principal component analysis (PCA) can improve the effective evaluation of potentially tolerant genotypes. Previous studies have shown that crop cultivars have significant genetic variability in their ability to withstand stresses, with features exhibited by both seedling shoots and roots [40,41,42]. Testing genotypes for salt and waterlogging tolerance early in growth has the potential to save time and resources because most crops are susceptible to combined stresses during this phase. Considering the above, our study aimed to evaluate the ability of 100 wheat genotypes to tolerate combined salt and waterlogging stresses during their seedling stage in order to find novel germplasm resources that are tolerant of these combined stresses. The study also sought to use genotypes selected using IS to gain a better understanding of their physio-biochemical characteristics, including Na^+^ and K^+^ accumulation, chlorophyll contents, cell membrane stability, and antioxidant enzyme activities that contribute to tolerance and susceptibility to these combined stresses. This knowledge will facilitate wheat breeding programs leading to the development of cultivars with enhanced resilience to combined stresses.

## 2. Results

### 2.1. Genotypic Differences in Response to Combined Salinity and Waterlogging Stresses

**Figure 1:**
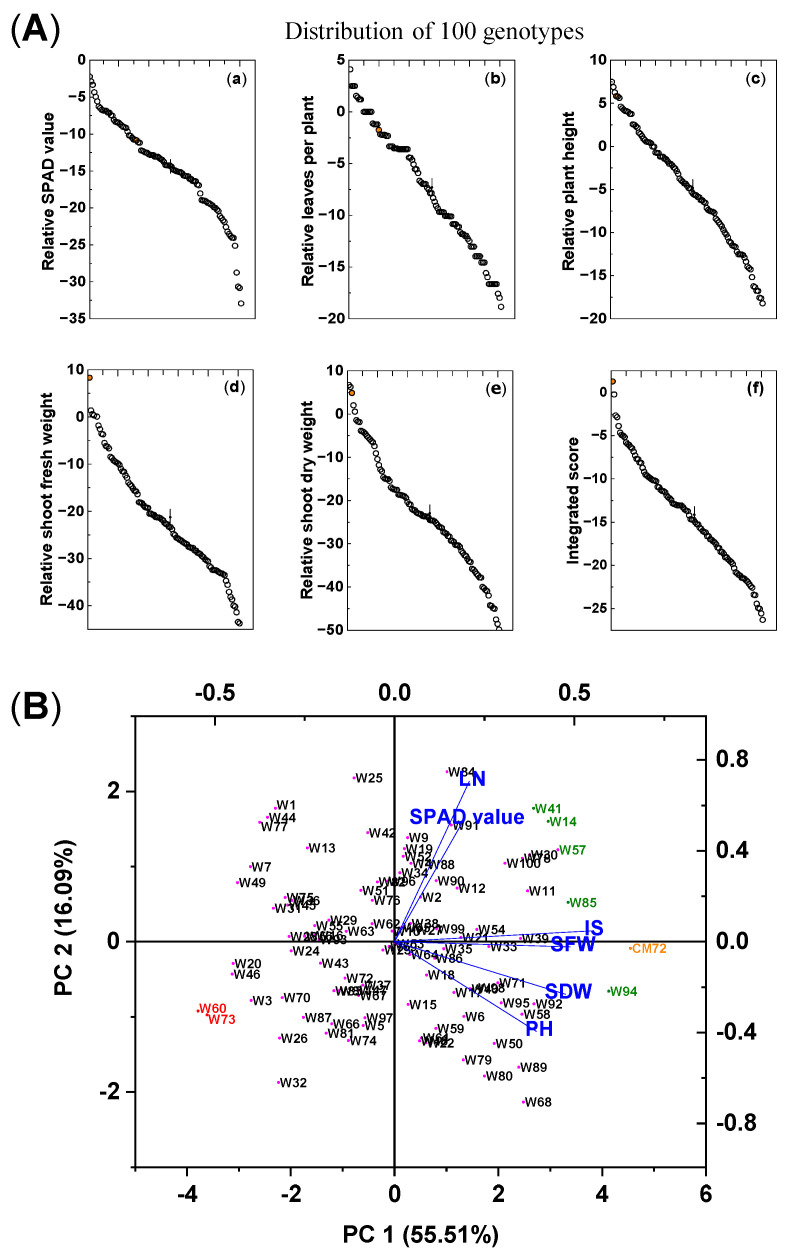
Differences in growth traits and integrated scores among 100 wheat varieties and CM72 (comparator barley genotype) subjected to combined 100 mM NaCl and waterlogging stresses. (**A**) Means of the relative differences (treatment/control) in various growth parameters (**a**–**f**) of five salinity/waterlogging-associated traits and their integrated score for each of the wheat genotypes and CM72 subjected to combined salt and waterlogging stresses. The orange symbol represents CM72. The vertical bars (“|”) represent LSD_0.05_ values between varieties. (**B**) Biplot of principal component analysis of individuals and explanatory variables based on data from the morpho-physiological traits of the 100 wheat genotypes and CM72 grown under combined salt and waterlogging stresses expressed as the percentage of control (%). The data are from the preliminary pot selection experiment and were measured 15 days after treatment. Data are presented as means of three biological replicates (each replicate contained four seedlings). Abbreviations: SPAD value, chlorophyll content; LN, number of leaves per plant; PH, plant height; SFW, shoot fresh weight; SDW, shoot dry weight; IS, integrated score. Green, red, and orange symbols relate the selected tolerant and sensitive wheat genotypes and CM72, respectively, based on their integrated scores.

The impact of the combined salinity (100 mM NaCl) and waterlogging (S + W) treatment on the growth of the 100 wheat genotypes and CM72 is shown in Table 1 and Figure 1A. Significant differences due to treatment were found among the genotypes in chlorophyll contents, leaf numbers (LN), plant heights (PH), and shoot fresh weights (SFW) and dry weights (SDW). These traits were reduced by 14.5%, 7.4%, 4.9%, 21.7%, and 23.1%, respectively, compared with the controls. For each trait, CM72 was placed at the high end of the distributions, and for SFW and integrated score (IS), it was higher than any of the wheat accessions; for the other traits, there were wheat accessions that exceeded the values for CM72. With respect to variation between genotypes, the coefficient of variation (CV) was highest for LN and PH, intermediate for the two measures of mass accumulation, and lowest for the IS and chlorophyll contents. The Shannon-Weaver diversity index was similar for chlorophyll contents, PH, SDW, and IS (2.06–2.04) but was lower (1.93–1.94) for LN and SFW.

### 2.2. Ranking of Wheat Genotypes for Combined Salt and Waterlogging Tolerance

Based on their IS rank, the following seven wheat genotypes were selected for further evaluation. The five most tolerant genotypes (highest IS) were Kzyl-Sark (W94), Misr4 (W85), Hofed (W57), BAW-1157 (W14), and Corack (W41), with IS values of −0.22, −2.66, −2.86, −3.88, and −4.65%, respectively. The most sensitive genotypes (lowest IS) were Livingstong (W73) and Sunvale (W60), with IS values of −26.32 and −25.58, respectively (Table S1). Principal component analysis (PCA) was used to aid the characterization and evaluation of these seven selected genotypes (Figure 1B). The first (PC1) and second (PC2) principal components explained 55.5% and 16.1% of the total variation among the genotypes, and the eigenvalues for all principal components are presented in Table S2. The five tolerant genotypes and CM72 are on the right of this ordination, and the two susceptible genotypes are on the left; the separation along the abscissa was mainly driven by PC1, and the explanatory variables, IS, SFW, SDW, and PH, contributed most to the separation along the abscissa. This separation confirms the ranking of the genotypes using the IS alone, and the seven selected wheat genotypes were used for further evaluation of their salt tolerance.

### 2.3. Hydroponic Salinity Validation Experiment

**Figure 2:**
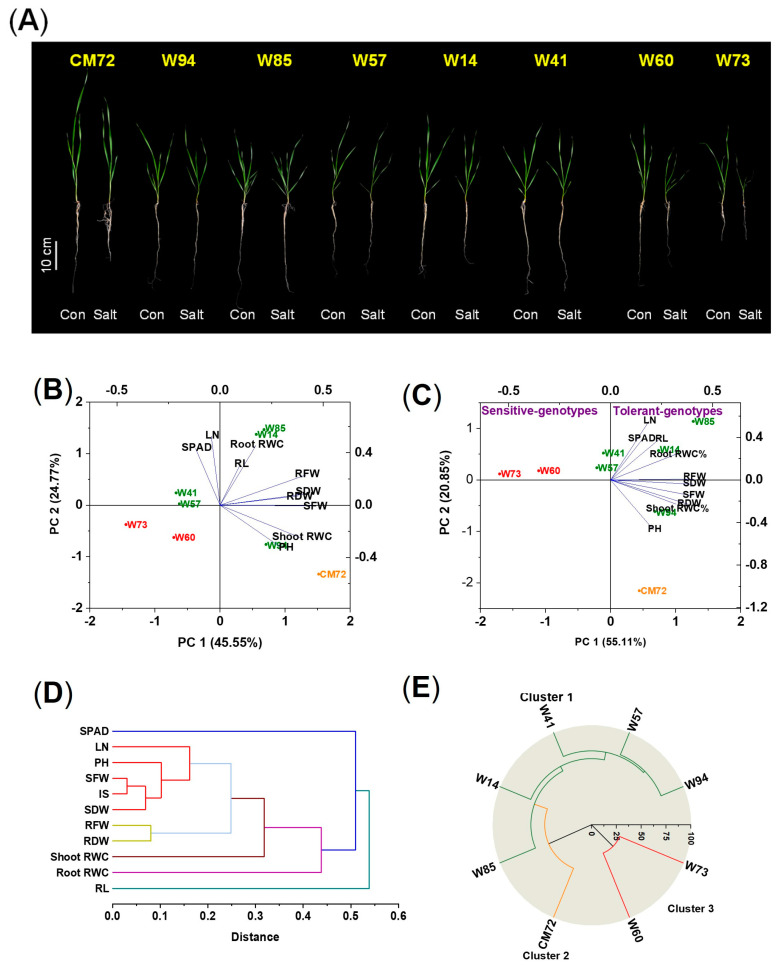
Phenotypes, principal component analysis, and hierarchical cluster analysis of the seedling in the hydroponic validation experiment of the five tolerant and two sensitive wheat genotypes and CM72 treated with and without the addition of 100 mM NaCl assessed 10 DAT. (**A**) Phenotypes of the seedlings of the five tolerant and two sensitive wheat genotypes and CM72. (**B**,**C**) Principal component analysis of data from plants grown under control (**B**) and salt stress (**C**) conditions. Green, red, and orange symbols represent tolerant and sensitive wheat genotypes and CM72, respectively. (**D**) Hierarchical cluster analysis of eight explanatory variables derived using the relative values of the morpho-physiological traits of the accessions. (**E**) Hierarchical cluster analysis of the seven selected genotypes and CM72. Three replicates were used (each replicate contained five seedlings). Abbreviations: SPAD, chlorophyll contents; LN, number of leaves per plant; PH, plant heights; RL, root lengths; SFW, shoot fresh weights; RFW, root fresh weights; SDW, shoot dry weights; RDW, root dry weights; RWC, relative water contents; IS, integrated scores.

In this experiment, the two sensitive and five tolerant wheat genotypes and CM72 were used to validate the accuracy of the screening under saline, hydroponic conditions; in this experiment, 10 morpho-physiological traits were measured 10 days after treatment (DAT). The impact of 100 mM NaCl on the measured traits is presented in Figure 2A and Figure S1 and Table S3. For the data as a whole, there were no significant differences among the eight genotypes due to the salinity treatment in RL and in shoot and root relative water contents (RWC); significant differences were found for all other traits. The IS of the tolerant wheat varieties were all larger than those of the sensitive ones, with CM72 being intermediate. Between the two sensitive genotypes, Sunvale (W73) was more affected by the salinity treatment than Livingstong (W60), displayed more severe symptoms (wilting and lodging), and had greater reductions in all traits except RL, root fresh weight (RFW), and root dry weight (RDW). The IS of the tolerant types ranked them as follows: Misr4 (W85) > Corack (W41) > Kzyl-Sark (W94) > Hofed (W57) > BAW-1157 (W14); the ranking is reflected in the magnitude of individual components of the IS. For most traits, the salinity treatment reduced the magnitude of the trait. However, for many of the tolerant wheat genotypes, RFW, RDW, and root RWC were increased in some tolerant genotypes due to salinity. For Misr4 (W85), there was also an increase in SDW in the salt-treated plants compared with the controls. For this genotype, no visual symptoms of salt stress appeared in the salt-treated plants.

The morpho-physiological characteristics of the seedlings were analyzed using PCA. The data from plants grown under control conditions showed little clustering with respect to salt tolerance; genotypes Corack (W41) and Hofed (W57) were categorized among the sensitive genotypes (Figure 2B). In contrast, the ordination of the data obtained under salt stress conditions clearly shows the separation of the genotypes with respect to tolerance/sensitivity (Figure 2C). The sensitive types are located to the extreme left of the ordination, close to the abscissa. The tolerant types are located close to the ordinate or to the right of the ordinate and, with the exception of Kzyl-Sark (W94), are above the abscissa. CM72 is towards the bottom of the lower right quadrant. Cluster analysis of the explanatory variables (Figure 2D) showed that chlorophyll content and RL were distinct from the other characters; shoot and root RWC were located close together, as were the two measures of biomass production; the remaining five variables all formed another group. Hierarchical cluster analysis performed on the experimental data categorized the chosen genotypes into three clusters (Figure 2E). The five wheat genotypes exhibiting tolerance clearly grouped into Cluster 1, CM72 formed Cluster 2, and the two sensitive genotypes constituted Cluster 3.

### 2.4. Further Evaluation of Misr4 (W85) and Sunvale (W73)

**Figure 3:**
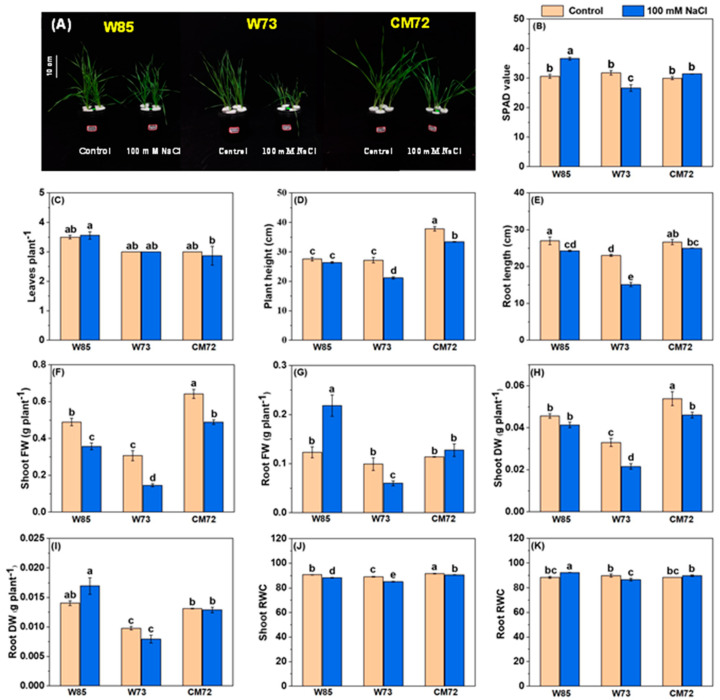
Morpho-physiological characteristics of the wheat genotypes Misr4 (W85)-tolerant, Sunvale (W73)-sensitive, and CM72. (**A**) seedlings phenotypes; (**B**–**K**) chlorophyll contents (SPAD values), leaves per plant, plant height, root length, shoot fresh weight, root fresh weight, shoot dry weight, root dry weight, shoot RWC and root RWC, respectively. The data were collected eight days after treatment with and without 100 mM NaCl. Values are means of three replicates (each replicate contained 10 seedlings), and the error bars are standard errors. Means annotated with the same letter are not statistically significantly different from each other according to least significant difference tests at *p* ≤ 0.05.

The results of the hydroponic selection experiment identified Sunvale (W73) as the most salt-sensitive wheat genotype and Misr4 (W85) as the most tolerant; the data relating to the effects of salt stress on their morpho-physiological characters are presented in Figure 3 and Table S4. Most surprising were the effects on chlorophyll contents, root and shoot FW, and root RWC (Figure 3B,G,I,K). For these characteristics, salt treatment tended to increase their magnitude in Misr4 (W85), reduce them in Sunvale (W73), and have little effect on CM72. The effects of the salt treatment on PH, RL, SFW, SDW and shoot RWC (Figure 3D–H,J) all had a similar pattern. The magnitudes of these characters tended to be lower due to the salt treatment in both the wheat genotypes, with the effects of salt being greater in Sunvale (W73) than in Misr4 (W85); the salt treatment also tended to reduce the magnitude of these characters in CM72, although to a lesser extent than for Sunvale (W73). Leaf numbers were little affected by salt treatment in any of the three genotypes.

#### 2.4.1. Accumulation of Na^+^ and K^+^

**Figure 4:**
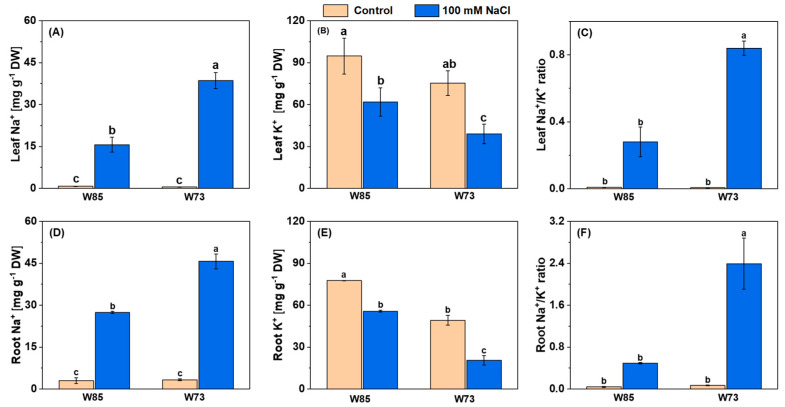
Leaf (**A**–**C**) and root (**D**–**F**) concentrations of Na^+^, K^+^, and the Na^+^/K^+^ ratio under control and salinity stress in Misr4 (W85; tolerant) and Sunvale (W73; sensitive) eight days after treatment with and without 100 mM NaCl. The data are means of three replicates, and the error bars are standard errors. Means annotated with the same letter are not significantly different from each other according to LSD tests at *p* ≤ 0.05.

The contents of Na^+^ in both leaves and roots are shown in Figure 4. Salt treatment increased Na^+^ concentrations in both organs, with the increases being greater in Sunvale (W73) than in Misr4 (W85) and in the roots compared with the shoots. With respect to K^+^, under control conditions, concentrations were slightly higher in Misr4 (W85) than in Sunvale (W73). Following treatment with NaCl, however, the K^+^ concentration was reduced in both genotypes and in both tissues. These changes in concentrations resulted in substantially higher Na^+^/K^+^ in the roots compared with the shoots and in Sunvale (W73) compared with Misr4 (W85).

#### 2.4.2. Photosynthetic Pigments

**Figure 5:**
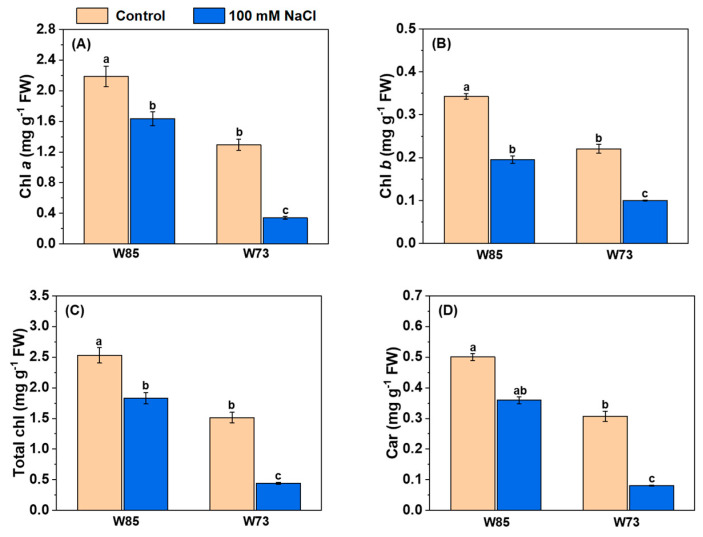
Effect of salinity stress on the leaf pigment contents of Misr4 (W85) and Sunvale (W73) five days after treatment with and without 100 mM NaCl. Panels (**A**–**D**) display chlorophyll a, b, total chlorophyll, and carotenoid contents (mg g^−1^ fresh weight) respectively. The data are means of three replicates, and the error bars are standard errors. Means annotated with the same letter are not significantly different from each other according to LSD tests at *p* ≤ 0.05.

All assessments of photosynthetic pigments showed the same patterns in their concentrations (Figure 5), irrespective of genotype. Under control conditions, the concentrations of all pigments were higher in Misr4 (W85) than in Sunvale (W73). Salt treatment reduced the concentrations of all pigments, with the reductions being greater in Sunvale (W73) than in Misr4 (W85).

#### 2.4.3. Reactive Oxygen Species, Malondialdehyde and Glutathione Concentrations, and Antioxidant Enzyme Activities

**Figure 6:**
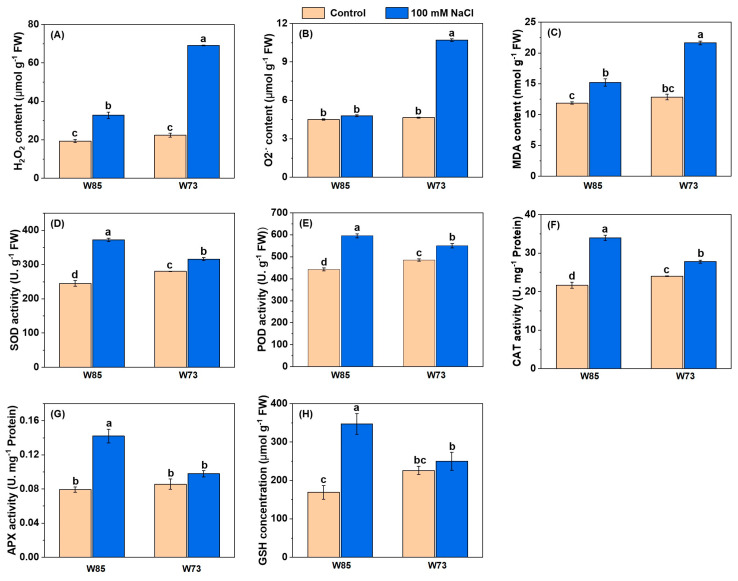
Contents of malondialdehyde (MDA) and glutathione (GSH), and antioxidant enzyme activities under control and salt stress in Misr4 (W85) and Sunvale (W73) five days after treatment with or without 100 mM NaCl. (**A**) Hydrogen peroxide (H_2_O_2_), (**B**) superoxide radical (O_2_^•−^), (**C**) malondialdehyde (MDA), (**D**) superoxide dismutase (SOD), (**E**) peroxidase (POD), (**F**) catalase (CAT), (**G**) ascorbate peroxidase (APX), and (**H**) glutathione (GSH). The data are means of three replicates, and the error bars are standard errors. Means annotated with the same letter are not significantly different from each other according to LSD tests at *p* ≤ 0.05.

As with the photosynthetic pigments, the concentrations or activities of ROS-associated compounds and enzymes (Figure 6) each showed similar patterns with respect to genotype and treatment. Under control conditions, all concentrations and activities were similar in the two genotypes or marginally higher in Sunvale (W73) than in Misr4 (W85). However, under treatment conditions, there were increases in all ROS-associated compounds and enzymes. Notably, concentrations of H_2_O_2_ and O_2_^•−^ in Sunvale (W73) were more than double those in Misr4 (W85). The activities of all the enzymes measured were higher in Misr4 (W85) than in Sunvale (W73), particularly for SOD and CAT. In addition, GSH concentrations approximately doubled in Misr4 (W85), whereas those in Sunvale (W73) were little affected by the treatment.

## 3. Discussion

The combination of salinity and waterlogging has severe adverse effects on the growth and survival of wheat [43,44,45], and the combined effects of these stresses are more detrimental than either stress imposed alone [1,2]. It is common for two or more abiotic stresses to have a greater negative effect on plants than when the stresses are applied individually [46]. Breeding for increased tolerance to these combined stresses is a key strategy to overcome these problems, and to facilitate breeding, a method for screening large numbers of genotypes is needed. Cereal crops are considered to be most sensitive to salt stress during both the vegetative and early stages of reproduction [47], and Ali et al. [48] showed that the tolerance of early-stage wheat seedlings correlates well with adult plant tolerance. Hence, the early growth stage is apposite for selecting salt- and waterlogging-tolerant genotypes. As a result, it is possible to quickly and efficiently evaluate large numbers of genotypes at this growth stage in the laboratory, thereby reducing the amount of work required in the field and the costs associated with this process [48]. Hence, in the preliminary screen of our study, young seedlings were used to quickly assess the tolerance of 100 wheat genotypes; this was combined with the measurement of multiple morpho-physiological characters to assess and rank the genotypes with regard to tolerance.

The large coefficients of variation of each of the traits measured in the preliminary screen suggest a rich genetic diversity exists among these 100 genotypes, and the distributions of these traits show their polygenic nature [49,50]. There was good agreement between the two techniques (ranking by IS and PCA) used in our study to evaluate the degree of tolerance or susceptibility of each genotype, and using these techniques, the five most tolerant and two most susceptible genotypes were chosen. The validation experiment and, to some extent, the final assessment of Misr4 (W85) and Sunvale (W73) confirmed the rankings obtained from the preliminary screen.

PCA is a multivariate method of assessment for inspecting large and complex datasets, and a biplot of the results may be used to determine variables that can partition data, based on their homogeneity and uniqueness, into groups and subgroups; hence, PCA can be used to aid the selection of parental material for breeding programs [48]. Our study’s PCA, based on relative values, identified distinct groups of germplasm accessions that were associated with either tolerance or sensitivity to the combined stresses. PCA has been utilized by numerous studies to identify grouping and diversity in wheat, both in the field [51] and at the seedling stage [52,53]. In addition, PCA can be used to aid the preservation of a wide range of genetic variability for future wheat breeding and production.

In the preliminary screen, the wheat genotypes differed greatly in growth traits when exposed to the combined stress, indicating the existence of useful genetic variation among genotypes. These differences in response are likely attributed to reduced stomatal conductance, suppression of metabolic processes, and heightened ROS production, which leads to oxygen-induced cellular damage [54]. However, some genotypes responded positively to the stress conditions, displaying greater plant heights, leaf growth, and biomass accumulation than the control group. Many studies, including those on important crop species, have also found similar increases in biomass production at certain Na^+^ concentrations [55,56], and increases have been reported for wheat [42,57,58]. The growth stimulation by Na^+^ in specific genotypes reported here and in other studies may be due to different genotypes having different Na^+^ to K^+^ ratios at which growth stimulation occurs. Sodium is classified as a functional nutrient [59] and, to some extent, is able to replace K, act as an osmoticium for cellular expansion, accompany cations during long-distance transport [59] and has a role in the regeneration of phosphoenolpyruvate [60]. The differences among the genotypes in their reaction to Na^+^ warrant further investigation.

The wheat genotypes assessed in the preliminary trial all had lower chlorophyll contents in the stress treatment than under control conditions, and they differed greatly in chlorophyll contents; many had relative chlorophyll contents greater than CM72. Reductions in Chl *a*, Chl *b*, and total chlorophylls were also found in the third trial, comparing Misr4 (W85) with Sunvale (W73). These changes are likely attributed to reduced chlorophyll biosynthesis and increased chlorophyll degradation; this topic is thoroughly reviewed by Li et al. [61]. Reduced chlorophyll contents are reported in wheat due to both waterlogging [62,63] and salinity [64,65]. Carotenoid contents were also markedly reduced in Sunvale (W73) and to a lesser extent in Misr4 (W85), and the reductions in carotenoids may also be due to lower carotenoid synthesis [66] and increased breakdown [67]. However, in the second validation trial, chlorophyll contents in four of the selected tolerant genotypes, including Misr4 (W85) and one of the two sensitive genotypes, Livingstong (W60), were increased. A meta-study by Agathokleous et al. [68] comprising 33 species and 20 stress-inducing agents found increases in chlorophyll contents but only at low-stress levels, and the authors suggest that this hormetic stimulation may pre-condition plants to future, larger environmental stresses. However, in our study, the reason for the increase in chlorophyll content in the validation trial but a reduction in the other two trials is unclear, as the same methodology was employed in trials 2 and 3, the same batches of seed were used throughout this study, and the plants were grown in the same growth chamber and are unlikely to be the effect of hormesis.

The inhibition of plant growth due to salinity and waterlogging may result from the adverse effects of increased Na^+^ and Cl^−^ levels alongside reduced K^+^ concentrations [69]. Kotula et al. [70] showed that oxygen shortages due to waterlogging disrupt the energy-dependent homeostasis of K^+^ and Na^+^ ions in barley roots, as energy-dependent ion discrimination at the root surface is harmed because energy production is lower. This means that less Na^+^ is excluded or more salt is absorbed [32,44]; this causes photosynthesis and shoot growth to slow significantly. The greater reduction in shoot growth compared with the reduction in the photosynthetic rate in the sensitive genotypes indicates a constraint on the utilization of photosynthates under combined stresses (Table S2). This could be due to lower water potentials and/or ionic toxicities that hinder the growth of root cells and, in turn, decrease overall plant biomass [71]. In the comparison of Misr4 (W85) with Sunvale (W73), Misr4 (W85) maintained a more normal Na^+^/K^+^ ratio than Sunvale (W73). Several studies have reported links between an ability to maintain, at least to some extent, a good ionic balance and tolerance to salinity and waterlogging [13,72,73], as found in our study. The maintenance of shoot Na^+^/K^+^ ratios is an important mechanism by which plants cope with stresses such as salinity and waterlogging [74,75,76]. When subjected to these stresses, reductions in growth occur due to increases in the accumulation of Na^+^ and decreases in the uptake and translocation of K^+^, resulting in perturbed Na^+^/K^+^ ratios [77,78,79]. Under the combination of saline and waterlogged conditions, the control of ion accumulation in the shoot can be further compromised [32,80]. This leads to additional, significant decreases in shoot growth [13,44], as energy-dependent ion discrimination at the root surface is reduced because of lower energy production [32,81]. In addition, Misr4 (W85) might use osmotic adjustment as an adaptive mechanism to maintain turgor pressure under stress conditions. An investigation of the association between this mechanism and the tolerance of wheat genotypes used in our study to the combined stresses may provide markers for use in breeding programs.

Cellular enzymatic and non-enzymatic antioxidant defense systems play a crucial role in protecting biological systems from the adverse effects of ROS. In the comparison of Misr4 (W85) with Sunvale (W73), Misr4 (W85) had higher SOD, POD, CAT, and APX activities and greater GSH concentrations than Sunvale (W73). As a result, Misr4 (W85) had lower concentrations of H_2_O_2_, O_2_^•−^, and MDA. These results are in agreement with the studies by Temel and Gozukirmizi [82], Feki et al. [83], Zeeshan et al. [31], and Kononenko et al. [53], who found that under salt stress, there were progressive increases in all the above enzymes in wheat plants. SOD catalytically converts O_2_^•−^ into H_2_O_2_, which is further catabolized by CAT to prevent oxidative damage. GSH also functions as a crucial component of the ascorbate-glutathione cycle. This cycle maintains reducing conditions by keeping the cellular concentration of reduced GSH high and the oxidized form, GSSG, low. The ease with which these activities and compounds can be measured makes them ideal candidates for inclusion in multifactorial assays of stress tolerance.

## 4. Materials and Methods

### 4.1. Preliminary Combined Salt and Waterlogging Screen

A pot experiment was conducted in a greenhouse at the Zijingang Campus, Zhejiang University, Hangzhou, China. A total of 100 wheat genotypes collected from different areas in the world (Table S5), including five from Egypt, seven from China, 36 from Bangladesh, and 52 from Australia, were used in this preliminary screen. In addition, the barley (*Hordeum vulgare* L.) cv. CM72 was grown because it is a salt- and waterlogging-tolerant cultivar [12]; we used CM72 because there are wheat varieties that are resistant to salinity or waterlogging stress but not to combined stresses. The soil used in this screen was collected from the university’s farm (depth 0–300 mm), air-dried, and sieved, and then 450 g aliquots were placed into plastic pots (height 120 mm, width 80 mm). Each pot was fertilized with 1 L of basal nutrient solution (BNS) [84]. Seeds were sown directly into each pot one week after the application of the BNS. Ten days post-emergence, the seedlings were thinned to five uniform plants per pot. Combined salinity and waterlogging treatments were applied to the seedlings at the 4-leaf stage to form two treatments: (1) control (non-salinized/waterlogged), in which soils in the pots were kept moist (60–80% water holding capacity) throughout, and (2) combined NaCl (100 mM) and waterlogging stress (S + W), in which the pots were placed into large plastic tanks (600 mm length × 400 mm width × 150 mm height), 24 pots per tank, and the solution levels in the tanks kept 20 mm above the soil surface to induce waterlogging for 15 days. Salt solutions were replaced every five days. The experiment was arranged in a randomized complete block design (RCBD) with three replications.

The chlorophyll contents of the seedlings were measured as SPAD values using a chlorophyll meter (Minolta Corporation, Ltd., Osaka, Japan) 15 days after treatment (DAT), according to Wu et al. [85]. Four seedlings were sampled 15 DAT for measurement of the following parameters: plant height (PH), shoot fresh weight (SFW) and shoot dry weight (SDW). Subsequently, the seedlings were gently uprooted and thoroughly rinsed with running tap water. After measuring numbers of leaves per plant (LN), the plants were separated into roots and shoots; shoot fresh weights (SFW) were measured immediately. The shoots were then dried at 75 °C for ~72 h until they reached constant weights, and shoot dry weights (SDW) were determined. From these data, an integrated score (IS) was calculated as follows:

The IS was calculated based on the percentage reduction in growth parameters relative to controls according to Foysal et al. [86] with some modifications using the following formula:

IS = [(SPAD value × 0.2) + (LN × 0.2) + (PH × 0.2) + (SFW × 0.2) + (SDW × 0.2)]

A less negative IS indicates a smaller negative impact of combined salinity and waterlogging stress and greater tolerance, whereas a more negative IS reflects a stronger negative impact from combined S + W stress and greater sensitivity. IS was used to determine the five most tolerant and two most sensitive wheat genotypes. A Shannon-Weaver diversity index (H’) was determined for each trait using the equation H’ = −Σ(pi × lnpi), where pi is the relative abundance of individual group of each accession examined and lnpi is the natural logarithm of the proportion value within each category [87].

### 4.2. Hydroponic Validation Experiment

Seven wheat genotypes were selected from the preliminary screen and used in a second experiment, along with CM72. Seeds from each genotype were germinated on filter papers within Petri dishes and placed in a plant growth chamber maintained at 23/18 °C (day/night) in the darkness for three days, followed by exposure to light for an additional four days. After this seven-day period, uniform seedlings from each genotype were selected and placed into foam plates with 105 uniformly distributed holes, one seedling per hole. Plastic containers (25 L) were then filled with BNS, on which the trays were floated. The experiment used three replicates per genotype (each replicate contained seven seedlings). The solution was continuously aerated and was renewed every five days. The pH of the solution was adjusted to 5.8 ± 0.2 with HCl or NaOH as required.

At the fourth leaf stage, seedlings were subjected to two treatments: BNS with the addition of 0 or 100 mM NaCl. Ten days after treatment, chlorophyll content, LN, PH, and RL were measured from five seedlings. The seedlings were then separated into shoots and roots to measure shoot and root FW, following which shoot and root DW were measured as described above. The relative water content (RWC) of both the shoots and roots was calculated utilizing the formula RWC = (FW − DW)/FW × 100 [88]. To assess and rank the salt tolerance of the wheat genotypes, an integrated score (IS) was used according to Foysal et al. [86] with some modifications as follows:

IS = [(SPAD value × 0.1) + (LN × 0.1) + (PH × 0.1) + (RL × 0.1) + (SFW × 0.1) + (RFW × 0.1) + (SDW × 0.1) + (RDW × 0.1) + (shoot RWC × 0.1) + (root RWC × 0.1)].

Based on IS rank, the genotypes exhibiting the highest tolerance and the greatest sensitivity to salt were selected and used for the work detailed in Section 4.3.

### 4.3. Evaluation of Misr4 (W85) and Sunvale (W73)

Wheat genotypes Misr4 (W85) and Sunvale (W73) were subjected to a further hydroponic study in a plant growth chamber. The growth and treatments condition were as in Section 2.2, except that there were three plants per hole. Five days after treatment, samples of the second leaves were frozen in liquid nitrogen and preserved at −80 °C to measure H_2_O_2_, O_2_^•−^, and MDA contents and the activities of SOD, POD, APX, CAT, and concentrations of GSH. Eight days after treatment, plants were sampled to determine morphological traits. Ten seedlings from each replicate were harvested and washed with pure water and dried using tissue paper; chlorophyll contents, LN, PH, RL, SFW, RFW, SDW, RDW, shoot RWC, and root RWC were measured as described above.

#### 4.3.1. Estimation of Na^+^ and K^+^ Concentrations

To measure Na^+^ and K^+^ concentrations, eight days after treatment, the dried leaf and root samples were ground to a powder and digested in 3 mL of nitric acid. The digestion process involved heating the samples in an aluminum block (Dry ThermoUnit DTU-2CN; TAITEC, Tokyo, Japan) at 100 °C for 2 h, followed by 120 °C for 3 h, and finally at 140 °C for 1 h. After digestion, the digests were diluted with deionized water. The concentrations of Na^+^ and K^+^ were determined using inductively coupled plasma-mass spectrometry (ICP-MS), according to Saeed et al. [89].

#### 4.3.2. Estimation of Photosynthetic Pigments

Samples (0.5 g) of wheat leaves taken five DAT were ground in 8 mL of 80% acetone (*v*/*v*). The homogenates were passed through filter paper, and then the absorbances of the resulting solutions were measured in a microplate reader at wavelengths of 645 nm and 663 nm for chlorophyll *a* and *b* and at 470 nm for carotenoids [90]. The calculations to determine concentrations of chlorophyll *a*, chlorophyll *b*, and carotenoids were performed using the formulas provided by [91]:

Chl *a* (mg g^−1^ FW) = [(13.95(OD_665_) − 6.88(OD_649_)] V/200 × W;

Chl *b* (mg g^−1^ FW) = [(24.96(OD_649_) − 7.32(OD_663_)] V/200 × W;

and

Car (mg g^−1^ FW) = [(1000(OD_470_) − (2.05(Chl *a*) − (114.80(Chl *b*)] V × 245/200 × W.

where Chl *a* = chlorophyll a, Chl *b* = chlorophyll b, Car = carotenoids, W refers to shoot weights (0.5 g), and V refers to acetone volumes (8 mL).

#### 4.3.3. Determination of Reactive Oxygen Species (H_2_O_2_ and O_2_^•−^), Lipid Peroxidation, Antioxidant Enzyme Activities, and Reduced Glutathione

Five days after treatment, fresh, fully expanded second leaves were collected, immediately frozen in liquid nitrogen, and preserved at −80 °C. The concentrations of H_2_O_2_ and O_2_^•−^ were measured according to Elstner and Heupel [92] and Yu et al. [93], respectively. The activities of SOD, POD, APX, CAT, and concentrations of GSH and MDA were measured with detection kits (Nanjing Jincheng Bioengineering Institute, Nanjing, China) according to the manufacturer’s instructions.

### 4.4. Statistical Analysis

Initial processing and analysis of the experimental data were conducted utilizing Excel 2023, following which statistical analyses were performed using Statistix v. 8.1 (Analytical Software, Tallahassee, FL, USA). The data from the preliminary screen was analyzed using one-way ANOVA. The least significant differences (LSD) at a significance level of 0.05 were employed to compare the mean values of each characteristic within each genotype. The data from the remaining experiments were analyzed by 2-way ANOVA. The software package Origin (Origin Lab 2021, version 9.1) was utilized to graph the results.

## 5. Conclusions

This study has shown that substantial variation exists in the expression of tolerance of the combined salinity and waterlogging stress among the genotypes of wheat examined. The relative tolerance of these genotypes was easily assessed through the use of a multifactorial, integrated scoring system based on morpho-physiological traits; the use of seedlings permitted multiple genotypes to be simultaneously evaluated. The application of PCA and hierarchical cluster analysis to examine wheat seedlings revealed strong correlations among many characteristics. These correlations may function as criteria for identifying wheat germplasm with salt and waterlogging tolerance. By combining the IS and PCA results, it was determined that the wheat genotypes Kzyl-Sark (W94), Misr4 (W85), Hofed (W57), BAW-1157 (W14), and Corack (W41), exhibited the highest levels of combined stress tolerance. The most tolerant genotype (W85; Misr4 from Egypt) and most susceptible genotype (W73; Sunvale from Australia) were assessed in depth. The higher tolerance of Misr4 (W85) was expressed through better ionic and redox homeostasis that resulted in higher contents of photosynthetic pigments. The tolerant genotypes identified can be used directly in breeding programs and for the assessment of further genotypes. The genotypes identified in our study provide ideal material for further physiological and molecular studies to determine any unique nature of the response to combined salinity and waterlogging stresses and how tolerance to this combination is controlled.

## Figures and Tables

**Table 1:** Relative effects of combined salt and waterlogging stresses on the agronomic traits of the 100 wheat genotypes and CM72 used in the preliminary screen after 15 days of treatment; relative values expressed as percentages of controls.

Reduction Percentage	SPAD Value	Leaves per Plant	Plant Height	Shoot FW	Shoot DW	Integrated Score ^a^
Maximum	−2.21	4.12	7.49	1.40	6.74	−0.22
Minimum	−32.95	−18.87	−18.24	−43.82	−49.89	−26.32
Mean	−14.45	−7.35	−4.93	−21.65	−23.08	−14.30
CM72	−10.87	−1.75	2.82	8.33	4.89	1.28
CV	24.31	42.15	44.52	36.27	33.98	24.94
Diversity index	2.05	1.93	2.06	1.94	2.04	2.05
Between genotypes	**	**	**	**	**	**

^a^ Integrated score = [(SPAD value × 0.2) + (LN × 0.2) + (PH × 0.2) + (SFW × 0.2) + (SDW × 0.2)]. The coefficient of variation (CV) and diversity index values were calculated after arcsine transformation of percentage data. ** = significance at *p* ≤ 0.01. For each genotype, three replicates were used (each replicate contained four seedlings).

## Data Availability

The original contributions presented in the study are included in the article; further inquiries can be directed to the corresponding author.

## Supplementary Materials

The following supporting information can be downloaded at https://www.mdpi.com/article/10.3390/plants14091268/s1, Figure S1: Effects of salinity stress on the five tolerant Kzyl-Sark (W94), Misr4 (W85), Hofed (W57), BAW-1157 (W14), and Corack (W41); two sensitive Livingstong (W60) and Sunvale (W73) genotypes; and CM72 in the hydroponic validation experiment with and without treatment with 100 mM NaCl. The plants were assessed 10 days after treatment, and the data are means ± SE (n = 3). Bars annotated with different letters indicate significant differences between means at *p* ≤ 0.05 according to least significance tests; Table S1: Chlorophyll contents (SPAD values), leaves per plant (LN), plant height (PH), shoot fresh weight (SFW) and dry weight (SDW), and the integrated score (IS) of CM72 (comparator genotype) and the selected tolerant (TG) and sensitive (SG) wheat genotypes after 15 days of combined salinity and waterlogging stresses (S+W) during the preliminary screen; Table S2: Eigenvalues of the correlation matrix of 100 wheat genotypes and CM72 (check genotype) after 15 days of exposure to 100 mM NaCl during the preliminary pot selection experiment based on relative values expressed as percentages of controls; Table S3: Relative (treatment/control) effects of combined salt and waterlogging stresses on the agronomic traits of CM72 and the five tolerant (TG) and two susceptible (SG) wheat genotypes in the hydroponic validation experiment; Table S4: Effect of salinity stress on chlorophyll contents (SPAD value), leaves per plant, plant height, root length, shoot fresh weight, root fresh weight, shoot and root biomass, and shoot and root relative water content and the integrated score of tolerant-genotype Misr4 (W85), sensitive-genotype Sunvale (W73), and CM72 (check genotype) in the validation experiment after 8 days of 100 mM NaCl expressed as the percentage of control; Table S5: Serial number, name, and country of origin of the 100 wheat genotypes used in the preliminary screen.

## References

[1] Anwar K., Joshi R., Dhankher O.P., Singla-Pareek S.L., Pareek A. (2021). Elucidating the Response of Crop Plants towards Individual, Combined, and Sequentially Occurring Abiotic Stresses. Int. J. Mol. Sci..

[2] Hussain S., Mehmood U., Ashraf U., Naseer M.A. (2022). Combined Salinity and Waterlogging Stress in Plants: Limitations and Tolerance Mechanisms. Climate Change and Crop Stress.

[3] Sorokin L.V., Mondello G. (2018). Entering the New +2 °C Global Warming Age and the Threat of World Ocean Expansion to Sustainable Economic Development. Climate Change, Extreme Events and Disaster Risk Reduction: Towards Sustainable Development Goals.

[4] Zaman M., Shahid S.A., Heng L., Shahid S.A., Zaman M., Heng L. (2018). Soil Salinity: Historical Perspectives and a World Overview of the Problem. Guideline for Salinity Assessment, Mitigation and Adaptation Using Nuclear and Related Techniques.

[5] Sui Y., Jiang R., Liu Y., Zhang X., Lin N., Zheng X., Li B., Yu H. (2025). Predicting the Spatial Distribution of Soil Salinity Based on Multi-Temporal Multispectral Images and Environmental Covariates. Comput. Electron. Agric..

[6] Shabala S. (2011). Physiological and Cellular Aspects of Phytotoxicity Tolerance in Plants: The Role of Membrane Transporters and Implications for Crop Breeding for Waterlogging Tolerance. New Phytol..

[7] Sachdev S., Ansari S.A., Ansari M.I., Fujita M., Hasanuzzaman M. (2021). Abiotic Stress and Reactive Oxygen Species: Generation, Signaling, and Defense Mechanisms. Antioxidants.

[8] Wu H.Y., Zhang P., Xu M., Zhuang L. (2018). Spatial-Temporal Variations of the Risk of Winter Wheat Loss Suffered from Spring Waterlogging Disaster in the Middle and Lower Yangtze River Reaches. Resour. Environ. Yangtze Basin.

[9] Pandey A.C., Singh S.K., Nathawat M.S. (2010). Waterlogging and Flood Hazards Vulnerability and Risk Assessment in Indo-Gangetic Plain. Nat. Hazards.

[10] Van Der Zee S., Stofberg S.F., Yang X., Liu Y., Islam M.N., Hu Y.F. (2017). Irrigation and Drainage in Agriculture: A Salinity and Environmental Perspective. Current Perspective on Irrigation and Drainage.

[11] Bakker D., Hamilton G., Hetherington R., Spann C. (2010). Salinity Dynamics and the Potential for Improvement of Waterlogged and Saline Land in a Mediterranean Climate Using Permanent Raised Beds. Soil Tillage Res..

[12] Falakboland Z., Zhou M., Zeng F., Kiani-Pouya A., Shabala L., Shabala S. (2017). Plant Ionic Relation and Whole-Plant Physiological Responses to Waterlogging, Salinity and Their Combination in Barley. Funct. Plant Biol..

[13] Malik A.I., English J.P., Colmer T.D. (2009). Tolerance of *Hordeum marinum* Accessions to O_2_ Deficiency, Salinity and these Stresses Combined. Ann. Bot..

[14] Renziehausen T., Frings S., Schmidt-Schippers R. (2024). “Against All Floods”: Plant Adaptation to Flooding Stress and Combined Abiotic Stresses. Plant J..

[15] Hasegawa P.M., Bressan R.A., Zhu J.-K., Bohnert H.J. (2000). Plant Cellular and Molecular Responses to High Salinity. Annu. Rev. Plant Biol..

[16] Zhu J.-K. (2001). Plant Salt Tolerance. Trends Plant Sci..

[17] Munns R. (2002). Comparative Physiology of Salt and Water Stress. Plant Cell Environ..

[18] Shabala S., Shabala L., Van Volkenburgh E., Newman I. (2005). Effect of Divalent Cations on Ion Fluxes and Leaf Photochemistry in Salinized Barley Leaves. J. Exp. Bot..

[19] McCord J.M. (2000). The Evolution of Free Radicals and Oxidative Stress. Am. J. Med..

[20] Jena N. (2012). DNA Damage by Reactive Species: Mechanisms, Mutation and Repair. J. Biosci..

[21] Das P., Agarwala N., Gill S.S., Varshney R.K. (2023). Emerging Role of Plant Long Non-coding RNAs (lncRNAs) in Salinity Stress Response. Plant Stress.

[22] Yu D., Zhou M., Chen W., Ding Z., Wang C., Qian Y., Liu Y., He S., Yang L. (2024). Characterization of Transcriptome Changes in Saline Stress Adaptation on *Leuciscus merzbacheri* using PacBio Iso-Seq and RNA-Seq. DNA Res..

[23] Zhao S., Zhang Q., Liu M., Zhou H., Ma C., Wang P. (2021). Regulation of Plant Responses to Salt Stress. Int. J. Mol. Sci..

[24] Foyer C.H., Noctor G. (2003). Redox Sensing and Signalling Associated with Reactive Oxygen in Chloroplasts, Peroxisomes and Mitochondria. Physiol. Plant..

[25] Bowler C., Montagu M.V., Inze D. (1992). Superoxide Dismutase and Stress Tolerance. Annu. Rev. Plant Physiol. Plant Mol. Biol..

[26] Willekens H., Inzé D., Van Montagu M., Van Camp W. (1995). Catalases in Plants. Mol. Breed..

[27] Caverzan A., Passaia G., Rosa S.B., Ribeiro C.W., Lazzarotto F., Margis-Pinheiro M. (2012). Plant Responses to Stresses: Role of Ascorbate Peroxidase in the Antioxidant Protection. Genet. Mol. Biol..

[28] Liang W., Ma X., Wan P., Liu L. (2018). Plant Salt-Tolerance Mechanism: A review. Biochem. Biophys. Res. Commun..

[29] Sharma P., Jha A.B., Dubey R.S., Pessarakli M. (2012). Reactive Oxygen Species, Oxidative Damage, and Antioxidative Defense Mechanism in Plants under Stressful Conditions. J. Bot..

[30] Dugasa M.T., Feng X., Wang N.-H., Wang J., Wu F. (2021). Comparative Transcriptome and Tolerance Mechanism Analysis in the Two Contrasting Wheat (*Triticum aestivum* L.) Cultivars in Response to Drought and Salinity Stresses. Plant Growth Regul..

[31] Zeeshan M., Lu M., Sehar S., Holford P., Wu F. (2020). Comparison of Biochemical, Anatomical, Morphological, and Physiological Responses to Salinity Stress in Wheat and Barley Genotypes Differing in Salinity Tolerance. Agronomy.

[32] Barrett-Lennard E. (2003). The Interaction between Waterlogging and Salinity in Higher Plants: Causes, Consequences and Implications. Plant Soil.

[33] Kunika B.K., Singh P.K., Rani V., Pandey G.C. (2019). Salinity Tolerance in Wheat: An Overview. Int. J. Chem. Stud..

[34] Tian L., Zhang Y., Chen P., Zhang F., Li J., Yan F., Dong Y., Feng B. (2021). How Does the Waterlogging Regime Affect Crop Yield? A Global Meta–Analysis. Front. Plant Sci..

[35] Erenstein O., Jaleta M., Mottaleb K.A., Sonder K., Donovan J., Braun H.-J. (2022). Global Trends in Wheat Production, Consumption and Trade. Wheat Improvement: Food Security in a Changing Climate.

[36] Liu K., Harrison M.T., Shabala S., Meinke H., Ahmed I., Zhang Y., Tian X., Zhou M. (2020). The State of the Art in Modeling Waterlogging Impacts on Plants: What Do We Know and What Do We Need to Know. Earth’s Future.

[37] Loutfy N., Sakuma Y., Gupta D.K., Inouhe M. (2020). Modifications of Water Status, Growth Rate and Antioxidant System in Two Wheat Cultivars as Affected by Salinity Stress and Salicylic Acid. J. Plant Res..

[38] Trnka M., Rötter R.P., Ruiz-Ramos M., Kersebaum K.C., Olesen J.E., Žalud Z., Semenov M.A. (2014). Adverse Weather Conditions for European Wheat Production Will Become More Frequent with Climate Change. Nat. Clim. Change.

[39] El-Hendawy S.E., Hu Y., Yakout G.M., Awad A.M., Hafiz S.E., Schmidhalter U. (2005). Evaluating Salt Tolerance of Wheat Genotypes Using Multiple Parameters. Eur. J. Agron..

[40] Hasan A., Hafiz H.R., Siddiqui N., Khatun M., Islam R., Mamun A.-A. (2015). Evaluation of Wheat Genotypes for Salt Tolerance Based on Some Physiological Traits. J. Crop Sci. Biotechnol..

[41] Chaurasia S., Kumar A., Singh A.K. (2022). Comprehensive Evaluation of Morpho-Physiological and Ionic Traits in Wheat (*Triticum aestivum* L.) Genotypes under Salinity Stress. Agriculture.

[42] Khan M.M., Rahman M.M., Hasan M.M., Amin M.F., Matin M.Q.I., Faruq G., Alkeridis L.A., Gaber A., Hossain A. (2024). Assessment of the Salt Tolerance of Diverse Bread Wheat (*Triticum aestivum* L.) Genotypes during the Early Growth Stage under Hydroponic Culture Conditions. Heliyon.

[43] Kononenko N.V., Lazareva E.M., Fedoreyeva L.I. (2023). Mechanisms of Antioxidant Resistance in Different Wheat Genotypes under Salt Stress and Hypoxia. Int. J. Mol. Sci..

[44] Saqib M., Akhatar J., Qureshi R.H. (2005). Na^+^ Exclusion and Salt Resistance of Wheat (*Triticum aestivum*) in Saline-Waterlogged Conditions Are Improved by the Development of Adventitious Nodal Roots and Cortical Root Aerenchyma. Plant Sci..

[45] Singh G., Kulshreshtha N., Singh B., Setter T.L., Singh M., Saharan M., Tyagi B., Verma A., Sharma I. (2014). Germplasm Characterization, Association and Clustering for Salinity and Waterlogging Tolerance in Bread Wheat (*Triticum aestivum*). Indian J. Agric. Sci..

[46] Choudhury F.K., Rivero R.M., Blumwald E., Mittler R. (2017). Reactive Oxygen Species, Abiotic Stress and Stress Combination. Plant J..

[47] Gerona M.E.B., Deocampo M.P., Egdane J.A., Ismail A.M., Dionisio-Sese M.L. (2019). Physiological Responses of Contrasting Rice Genotypes to Salt Stress at Reproductive Stage. Rice Sci..

[48] Ali Z., Salam A., Azhar F.M., Khan I.A., Khan A.A., Bahadur S., Mahmood T., Ahmad A., Trethowan R. (2012). The Response of Genetically Distinct Bread Wheat Genotypes to Salinity Stress. Plant Breed..

[49] Baloglu M.C., Oz M.T., Oktem H.A., Yucel M. (2012). Expression Analysis of *TaNAC69-1* and *TtNAMB-2*, Wheat NAC family Transcription Factor Genes Under Abiotic Stress Conditions in Durum Wheat (*Triticum turgidum*). Plant Mol. Biol. Rep..

[50] Barghi N., Hermisson J., Schlötterer C. (2020). Polygenic Adaptation: A Unifying Framework to Understand Positive Selection. Nat. Rev. Genet..

[51] Sisodia B., Rai V. (2017). An Application of Principal Component Analysis for Pre-Harvest Forecast Model for Wheat Crop Based on Biometrical Characters. Int. Res. J. Agric. Econ..

[52] Hussain S., Khaliq A., Matloob A., Wahid M.A., Afzal I. (2013). Germination and Growth Response of Three Wheat Cultivars to NaCl Salinity. Soil Environ..

[53] Uzair M., Ali M., Fiaz S., Attia K., Khan N., Al-Doss A.A., Khan M.R., Ali Z. (2022). The Characterization of Wheat Genotypes for Salinity Tolerance Using Morpho-Physiological Indices under Hydroponic Conditions. Saudi J. Biol. Sci..

[54] Neill S.J., Desikan R., Clarke A., Hurst R.D., Hancock J.T. (2002). Hydrogen Peroxide and Nitric Oxide as Signalling Molecules in Plants. J. Exp. Bot..

[55] Kronzucker H.J., Coskun D., Schulze L.M., Wong J.R., Britto D.T. (2013). Sodium as Nutrient and Toxicant. Plant Soil.

[56] Sreesaeng J., Qiu C.-W., Zhang S., Shi S.-H., Luo L., Holford P., Wu F. (2024). Identification and Characterization of Hull-less Barley (*Hordeum vulgare* L.) Germplasms for Salt Tolerance. Plant Growth Regul..

[57] Box S., Schachtman D.P. (2000). The Effect of Low Concentrations of Sodium on Potassium Uptake and Growth of Wheat. Funct. Plant Biol..

[58] Krishnasamy K., Bell R., Ma Q. (2014). Wheat Responses to Sodium Vary with Potassium Use Efficiency of Cultivars. Front. Plant Sci..

[59] Subbarao G., Ito O., Berry W., Wheeler R. (2003). Sodium-A Functional Plant Nutrient. Crit. Rev. Plant Sci..

[60] Murata S., Kobayashi M., Matoh T., Sekiya J. (1992). Sodium Stimulates Regeneration of Phospho Enol Pyruvate in Mesophyll Chloroplasts of *Amaranthus tricolor*. Plant Cell Physiol..

[61] Li X., Zhang W., Niu D., Liu X. (2024). Effects of Abiotic Stress on Chlorophyll Metabolism. Plant Sci..

[62] Herzog M., Striker G.G., Colmer T.D., Pedersen O. (2016). Mechanisms of Waterlogging Tolerance in Wheat–A Review of Root and Shoot Physiology. Plant Cell Environ..

[63] Ozcubukcu S., Ergun N. (2013). Effects of Waterlogging and Nitric Oxide on Chlorophyll and Carotenoid Pigments of Wheat. J. Food Agric. Environ..

[64] Khan A.M., Shirazi M.U., Khan M.A., Mujtaba S.M., Islam E., Mumtaz S., Shereen A., Ansari R.U., Ashraf M.Y. (2009). Role of Proline, K/Na Ratio and Chlorophyll Content in Salt Tolerance of Wheat (*Triticum aestivum* L.). Pak. J. Bot..

[65] Saddiq M.S., Iqbal S., Hafeez M.B., Ibrahim A.M., Raza A., Fatima E.M., Baloch H., Jahanzaib, Woodrow P., Ciarmiello L.F. (2021). Effect of Salinity Stress on Physiological Changes in Winter and Spring Wheat. Agronomy.

[66] Tsai Y.-C., Chen K.-C., Cheng T.-S., Lee C., Lin S.-H., Tung C.-W. (2019). Chlorophyll Fluorescence Analysis in Diverse Rice Varieties Reveals the Positive Correlation between the Seedlings’ Salt Tolerance and Photosynthetic Efficiency. BMC Plant Biol..

[67] Ramel F., Mialoundama A.S., Havaux M. (2013). Nonenzymic Carotenoid Oxidation and Photooxidative Stress Signalling in Plants. J. Exp. Bot..

[68] Agathokleous E., Feng Z., Peñuelas J. (2020). Chlorophyll Hormesis: Are Chlorophylls Major Components of Stress Biology in Higher Plants?. Sci. Total Environ..

[69] Barrett-Lennard E.G., Shabala S.N. (2013). The Waterlogging/Salinity Interaction in Higher Plants Revisited-Focusing on the Hypoxia-Induced Disturbance to K^+^ Homeostasis. Funct. Plant Biol..

[70] Kotula L., Clode P.L., Striker G.G., Pedersen O., Läuchli A., Shabala S., Colmer T.D. (2015). Oxygen Deficiency and Salinity Affect Cell-Specific Ion Concentrations in Adventitious Roots of Barley (*Hordeum vulgare*). New Phytol..

[71] Ibrahimova U., Kumari P., Yadav S., Rastogi A., Antala M., Suleymanova Z., Zivcak M., Tahjib-Ul-Arif M., Hussain S., Abdelhamid M. (2021). Progress in Understanding Salt Stress Response in Plants Using Biotechnological Tools. J. Biotechnol..

[72] Schachtman D., Munns R. (1992). Sodium Accumulation in Leaves of *Triticum* Species That Differ in Salt Tolerance. Funct. Plant Biol..

[73] Saqib M., Akhtar J., Qureshi R.H., Nasim M. (2013). Selection and Characterization of Wheat Genotypes for Saline Soils Prone to Waterlogging. J. Plant Nutr. Soil Sci..

[74] Assaha D.V., Ueda A., Saneoka H., Al-Yahyai R., Yaish M.W. (2017). The Role of Na^+^ and K^+^ Transporters in Salt Stress Adaptation in Glycophytes. Front. Physiol..

[75] Katschnig D., Bliek T., Rozema J., Schat H. (2015). Constitutive High-Level SOS1 Expression and Absence of HKT1; 1 Expression in the Salt-Accumulating Halophyte *Salicornia dolichostachya*. Plant Sci..

[76] Munns R., Tester M. (2008). Mechanisms of Aalinity Tolerance. Annu. Rev. Plant Biol..

[77] Fortmeier R., Schubert S. (1995). Salt Tolerance of Maize (*Zea mays* L.): The Role of Sodium Exclusion. Plant Cell Environ..

[78] SüMER A., Zörb C., Yan Feng Y.F., Schubert S. (2004). Evidence of Sodium Toxicity for the Vegetative Growth of Maize (*Zea mays* L.) during the First Phase of Salt Stress. J. Appl. Bot. Food Qual..

[79] Tester M., Davenport R. (2003). Na^+^ Tolerance and Na^+^ Transport in Higher Plants. Ann. Bot..

[80] Drew M., Guenther J., Läuchli A. (1988). The Combined Effects of Salinity and Root Anoxia on Growth and Net Na^+^ and K^+^-Accumulation in *Zea mays* Grown in Solution Culture. Ann. Bot..

[81] Gill M.B., Zeng F., Shabala L., Böhm J., Zhang G., Zhou M., Shabala S. (2018). The Ability to Regulate Voltage-Gated K^+^-Permeable Channels in the Mature Root Epidermis Is Essential for Waterlogging Tolerance in Barley. J. Exp. Bot..

[82] Temel A., Gozukirmizi N. (2015). Physiological and Molecular Changes in Barley and Wheat Under Salinity. J. Appl. Biochem. Biotechnol..

[83] Feki K., Tounsi S., Masmoudi K., Brini F. (2017). The Durum Wheat Plasma Membrane Na^+^/H^+^ Antiporter SOS1 Is Involved in Oxidative Stress Response. Protoplasma.

[84] Wu F., Zhang G., Yu J. (2003). Interaction of Cadmium and Four Microelements for Uptake and Translocation in Different Barley Genotypes. Commun. Soil Sci. Plant Anal..

[85] Wu F., Wu L., Xu F. (1998). The Chlorophyll Meter to Predicting Nitrogen Sidedress Requirements for Short-Season Cotton (*Gossypium hirsutum* L.). Field Crops Res..

[86] Foysal M.R.A., Qiu C.W., Sreesaeng J., Elhabashy S., Akhter D., Zhang S., Shi S.H., Wu F. (2024). Comprehensive Physio-Biochemical Evaluation Reveals Promising Genotypes and Mechanisms for Cadmium Tolerance in Tibetan Hull-Less Barley. Plants.

[87] Shannon C.E., Weaver W. (1949). The Mathematical Theory of Communication.

[88] Nxele X., Klein A., Ndimba B. (2017). Drought and Salinity Stress Alters ROS Accumulation, Water Retention, and Osmolyte Content in Sorghum Plants. S. Afr. J. Bot..

[89] Saeed M., Zhao H., Chen Z., Ju P., Wang G., Zhou C., Jia H., Zhu C., Jia H., Jiao Y. (2024). Wax Bayberry as a Suitable Rootstock for Chinese Red Bayberry in Saline-Alkali Soil. Sci. Hortic..

[90] Arnon D.I. (1949). Copper Enzymes in Isolated Chloroplasts. Polyphenoloxidase in *Beta vulgaris*. Plant Physiol..

[91] Knight S.L., Mitchell C.A. (1983). Enhancement of Lettuce Yield by Manipulation of Light and Nitrogen Nutrition. J. Am. Soc. Hortic. Sci..

[92] Elstner E.F., Heupel A. (1976). Inhibition of Nitrite Formation from Hydroxylammonium Chloride: A Simple Assay for Superoxide Dismutase. Anal. Biochem..

[93] Yu C.-W., Murphy T.M., Lin C.-H. (2003). Hydrogen Peroxide-Induced Chilling Tolerance in Mung Beans Mediated through ABA-Independent Glutathione Accumulation. Funct. Plant Biol..

