# Supplementary material for: Identification of Wheat Genotypes with High Tolerance to Combined Salt and Waterlogging Stresses Using Biochemical and Morpho-Physiological Insights at the Seedling Stage
Source: Plants (Basel). 2025 Apr 22;14(9):1268. doi: 10.3390/plants14091268 (PMC12073303; doi:10.3390/plants14091268)
Supplement: Supplementary file 1 [file plants-14-01268-s001.zip › plants-3567769-Supplementary.pdf]

Supplementary

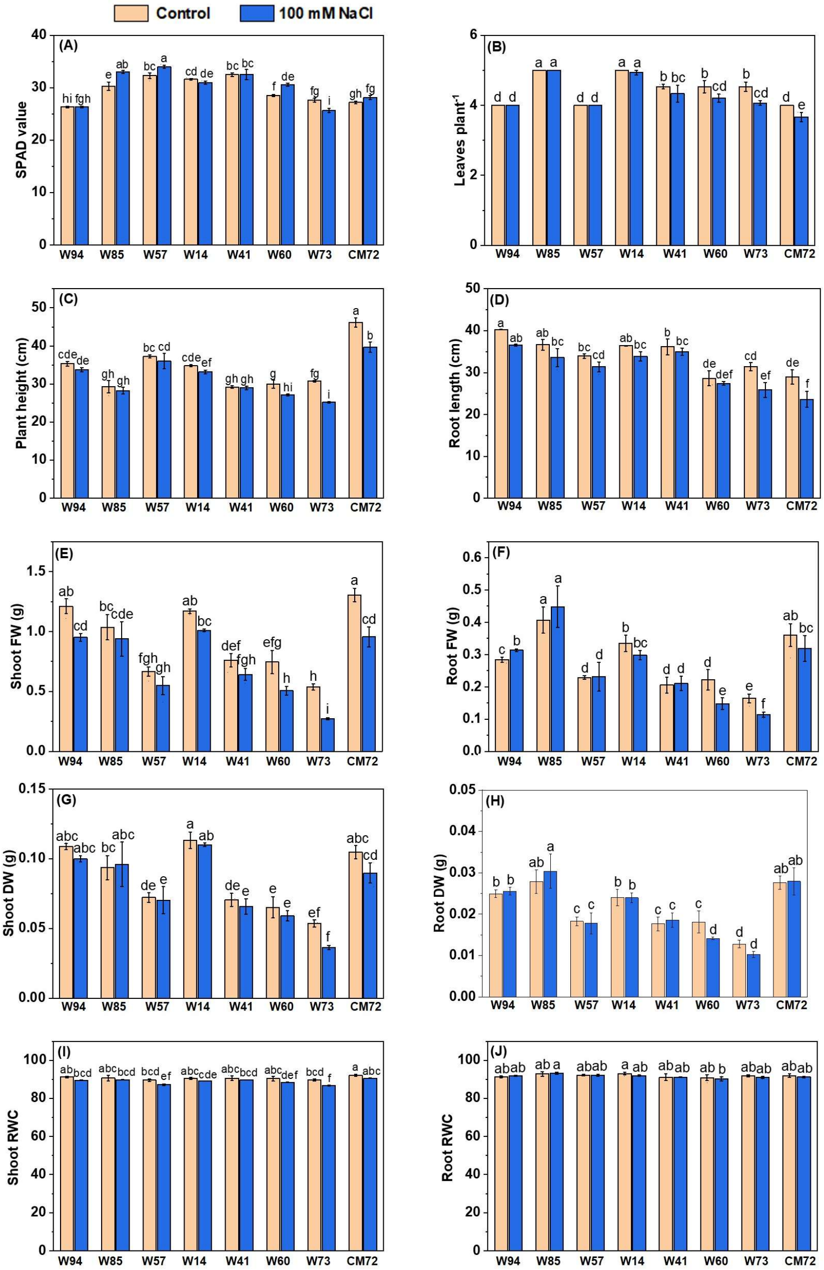

Figure S1. Effects of treatment with and without with 100 mM NaCl salinity stress on the five tolerant (Kzyl-Sark (W94), Misr4 (W85), Hofed (W57), BAW-1157 (W14) and Corack (W41)), two sensitive (Livingstong (W60) and Sunvale (W73)), and CM72 in the hydroponic validation experiment. The plants were assessed 10 days after treatment, and the data are means ± SE (n = 3). Bars annotated with different letters indicate significant differences between means at $p \leq 0.05$ according to least significance tests.

Table S1. Chlorophyll contents (SPAD values), leaves per plant (LN), plant height (PH), shoot fresh (SFW) and dry weight (SDW), and the integrated score (IS) of CM72 (comparator genotype) and the selected tolerant (TG) and sensitive (SG) wheat genotypes after 15 days of combined salinity and waterlogging stresses (S+W) during in the preliminary screen.

Genotypes	SPAD value			Leaves per plant			Plant height (mm)			Shoot FW (g plant^{-1})			Shoot DW (g plant^{-1})			Integrated score ^{a}
	CK	S+W	Reduction	CK	S+W	Reduction	CK	S+W	Reduction	CK	S+W	Reduction	CK	S+W	Reduction	
**CM72**	27.60^{e}	24.60^{e}	(-10.87) ^{b}[1]	3.80^{e}	3.37^{d}	(-1.75) ^{ab}	178.0^{b}	188.3^{ab}	(5.82) ^{a}	0.548^{cd}	0.631^{ab}	(8.33) ^{a}	0.116^{b}	0.127^{a}	(4.89) ^{ab}	1.28^{a}
<u>Tolerant genotypes</u>																
**W94**	36.15^{b}	33.13^{b}	(-8.34) ^{ab}	5.00^{ab}	4.63^{ab}	(-7.33) ^{bc}	135.0^{de}	145.1^{de}	(7.49) ^{a}	0.595^{bc}	0.597^{ab}	(0.34) ^{ab}	0.129^{b}	0.137^{a}	(6.74) ^{a}	-0.22^{ab}
**W85**	32.70^{c}	29.75^{c}	(-9.02) ^{ab}	5.00^{ab}	5.00^{a}	(0.00) ^{ab}	145.5^{d}	151.0^{cde}	(3.78) ^{a}	0.518^{d}	0.479^{b}	(-6.18) ^{b}	0.099^{c}	0.097^{b}	(-1.86) ^{abc}	-2.66^{ab}
**W57**	40.5^{a}	37.00^{a}	(-8.64) ^{ab}	4.30^{d}	4.37^{bc}	(1.55) ^{ab}	177.5^{b}	170.5^{bc}	(-3.94) ^{ab}	0.484^{d}	0.487^{b}	(0.60) ^{ab}	0.096^{c}	0.093^{b}	(-3.86) ^{abc}	-2.86^{ab}
**W14**	30.10^{d}	29.43^{c}	(-2.21) ^{a}	4.50^{cd}	4.50^{b}	(0.00) ^{ab}	191.2^{a}	192.1^{a}	(0.49) ^{a}	0.609^{bc}	0.619^{a}	(1.40) ^{ab}	0.156^{a}	0.126^{a}	(-19.06) ^{cde}	-3.88^{ab}
**W41**	35.20^{b}	32.83^{b}	(-6.72) ^{ab}	4.45^{cd}	4.63^{ab}	(4.12) ^{a}	165.3^{c}	162.0^{cd}	(-2.02) ^{a}	0.706^{a}	0.680^{ab}	(-3.69) ^{ab}	0.145^{a}	0.123^{a}	(-14.94) ^{bcd}	-4.65^{b}
<u>Sensitive genotypes</u>																
**W60**	35.50^{b}	28.57^{cd}	(-19.53) ^{c}	5.30^{a}	4.30^{bc}	(-18.87) ^{d}	167.0^{bc}	139.7^{e}	(-16.37) ^{bc}	0.629^{b}	0.377^{b}	(-40.01) ^{d}	0.123^{b}	0.078^{b}	(-36.82) ^{e}	-26.32^{c}
**W73**	39.45^{a}	26.45^{de}	(-32.95) ^{d}	4.80^{bc}	4.00^{cd}	(-16.67) ^{cd}	130.7^{e}	106.8^{f}	(-18.24) ^{c}	0.383^{e}	0.284^{b}	(-25.78) ^{c}	0.078^{d}	0.051^{c}	(-34.26) ^{de}	-25.58^{c}
**Mean of 7 genotypes**	35.23	31.02	(-14.49)	4.76	4.49	(-5.31)	158.9	152.5	(-4.12)	0.561	0.503	(-10.47)	0.118	0.100	(-14.87)	-9.45
**Mean of 5 TG**	34.33	32.43	(-6.99)	4.65	4.63	(-0.33)	162.9	164.1	(1.16)	0.582	0.572	(-1.51)	0.125	0.115	(-6.60)	-2.85
**Mean of 2 SG**	37.48	27.51	(-26.24)	5.05	4.15	(-17.77)	148.9	123.3	(-17.31)	0.506	0.331	(-32.90)	0.101	0.065	(-35.54)	-25.95
**$LSD_{0.05}$**	1.94	2.45	7.14	0.41	0.45	9.96	1.21	2.14	13.47	0.067	0.846	14.16	0.015	0.022	20.15	5.53

[1] Values within brackets represent the relative reduction in S+W treatment compared to the control. ^{a} Integrated score = [(SPAD value × 0.2) + (LN × 0.2) + (PH × 0.2) + (SFW × 0.2) + (SDW × 0.2)]. For each genotype, three replicates were used (each replicate contained 4 seedlings). Within column, means annotated with the same letter are not significantly different from each other according to least significance tests at $p \leq 0.05$.

Table S2. Eigenvalues of the correlation matrix of 100 wheat genotypes and CM72 (check genotype) after 15 days of exposure to 100 mM NaCl during the preliminary pot selection experiment based on relative values expressed as percentages of controls.

PC	Percentage			Characters	Coefficients[a]			
	Eigenvalue	Variance %	Cumulative %		PC1	PC2	PC3	PC4
**1**	3.331	55.51	55.51	SPAD	0.193	0.538	0.788	0.061
**2**	0.965	16.09	71.60	PH	0.208	0.706	-0.596	0.277
**3**	0.948	15.79	87.40	LN	0.394	-0.395	0.086	0.804
**4**	0.501	8.35	95.75	SFW	0.489	-0.023	-0.004	-0.376
**5**	0.255	4.25	100.00	SDW	0.476	-0.231	-0.130	-0.359
**6**	0.000	0.00	100.00	IS	0.547	0.048	0.002	-0.058

Abbreviations: SPAD, chlorophyll contents; PH, plant height; LN, number of leaves per plant; SFW, shoot fresh weight; SDW, shoot dry weight; IS, integrated score. [a]The coefficients are the correlations between the variables (e.g., PH and SPAD value) and the principal components, PC1, PC2, PC3, PC4.

Table S3. Relative (treatment/control) effects of combined salt and waterlogging stresses on the agronomic traits of CM72, and the five tolerant (TG) and two susceptible (SG) wheat genotypes in the hydroponic validation experiment after 10 days of treatment, expressed as the percentage of control.

Genotypes	SPAD value	Leaves per plant	Plant height	Root length	Shoot FW	Root FW	Shoot DW	Root DW	Shoot RWC	Root RWC	Integrated score
CM72	+3.47[bc]	-8.33[c]	-14.07[b]	-18.47 [ns]	-27.05[bc]	-7.85[ab]	-14.58[ab]	+1.95[ab]	-1.61[ns]	-0.71[ns]	-8.72[abc]
						<u>**Five tolerant genotypes**</u>					
W94	+3.68[bc]	0.00[a]	-4.42[a]	-9.36 [ns]	-21.16[abc]	+10.36[a]	-8.06[a]	+2.42[ab]	-1.91[ns]	+0.65[ns]	-2.78[ab]
W85	+9.16[a]	0.00[a]	-3.13[a]	-8.10 [ns]	-9.66[a]	+11.00[a]	+2.02[a]	+9.36[a]	-0.86[ns]	+0.41[ns]	+1.02[a]
W57	+5.09[ab]	0.00[a]	-3.26[a]	-7.63 [ns]	-18.34[abc]	+0.51[ab]	-3.62[a]	-3.70[ab]	-2.61[ns]	+0.00[ns]	-3.36[ab]
W14	-2.11[d]	-1.33[ab]	-4.58[a]	-6.83 [ns]	-13.66[ab]	-10.24[ab]	-5.25[a]	+0.58[ab]	-1.04[ns]	-1.16[ns]	-4.56[ab]
W41	+0.08[cd]	-4.48[abc]	-0.62[a]	-2.96 [ns]	-15.12[ab]	+3.47[a]	-6.26[a]	+6.18[a]	-0.87[ns]	+0.12[ns]	-2.05[ab]
						<u>**Two sensitive genotypes**</u>					
W60	+7.13[ab]	-7.26[bc]	-9.37[ab]	-3.56 [ns]	-31.04[c]	-32.57[b]	-8.36[a]	-19.37[b]	-2.19[ns]	-0.68[ns]	-10.73[bc]
W73	-7.13[e]	-10.23[c]	-18.09[b]	-17.22 [ns]	-48.92[d]	-30.16[b]	-31.61[b]	-19.23[b]	-3.30[ns]	-0.89[ns]	-18.68[c]
Mean of all 7 genotypes	+2.27	-3.33	-6.21	-7.95	-22.56	-6.80	-8.73	-3.39	-1.83	-0.22	-5.88
Mean of 5 TG	+3.18	-1.16	-3.20	-6.98	-15.59	+3.02	-4.23	+2.97	-1.46	0.00	-2.34
Mean of 2 SG	+0.00	-8.75	-13.73	-10.39	-39.98	-31.36	-19.99	-19.30	-2.74	-0.78	-14.70
$LSD_{0.05}$	4.86	6.8	9.42	ns	15.09	33.37	19.43	25.41	ns	ns	10.19

Values are expressed as a reduced (-)/increased (+) percentages compared the controls. Within a column, means followed by the same letter(s) are not significantly different according to LSD tests at $p \leq 0.05$. ns: not significant. For each genotype, three replicates were used (each replicate contained 5 seedlings).

Table S4. Effect of salinity stress on chlorophyll contents (SPAD value), leaves per plant, plant height, root length, shoot fresh weight, root fresh weight, shoot and root biomass, and shoot and root relative water content and the integrated score of tolerant-genotype Misr4 (W85), sensitive-genotype Sunvale (W73), and CM72 (check genotype) in the validation experiment after 8 days of 100 mM NaCl expressed as the percentage of control.

Genotypes	SPAD value	Leaves per plant	Plant height	Root length	Shoot FW	Root FW	Shoot DW	Root DW	Shoot RWC	Root RWC	Integrated score
**W85**	19.79[a]	1.57[a]	-4.01[a]	-9.85[a]	-26.71[a]	77.04[a]	-9.29[a]	20.79[a]	-2.49[a]	4.26[a]	7.11[a]
**W73**	-16.18[b]	0.00[a]	-21.88[b]	-34.32[b]	-51.62[b]	-38.28[c]	-34.08[a]	-18.93[b]	-4.41[b]	-3.47[b]	-22.32[b]
**CM72**	4.96[c]	-4.44[a]	-11.53[ab]	-5.98[a]	-23.59[a]	12.31[b]	-13.51[a]	-2.00[ab]	-1.15[a]	1.51[a]	-4.34[a]
**Between varieties**	**	NS	*	**	*	**	NS	*	**	**	**
**$LSD_{0.05}$**	8.08	24.23	13.75	13.84	21.93	36.09	25.97	28.24	1.58	3.18	12.38

* and **, significant at 0.05 and 0.01 levels of probability, respectively, between varieties under 100 mM NaCl. NS, not significant. For each genotype, three replicates were used (each replicate contained 10 seedlings).

Table S5. Serial number, name and country of origin of the 100 wheat genotypes used in the preliminary screen.

Sr. No.	Name	Origin	Sr. No.	Name	Origin	Sr. No.	Name	Origin
**W1**	BARI-Gom-22	Bangladesh	**W42**	CUNNINGHAM	Australia	**W83**	Sunmate	Australia
**W2**	BARI-Gom-23	Bangladesh	**W43**	DIAMONDBIRD	Australia	**W84**	Misr3	Egypt
**W3**	BARI-Gom-24	Bangladesh	**W44**	DRYSDALE	Australia	**W85**	Misr4	Egypt
**W4**	BARI-Gom-25	Bangladesh	**W45**	EGA BONNIE ROCK	Australia	**W86**	Sids14	Egypt
**W5**	BARI-Gom-28	Bangladesh	**W46**	EGA BOUNTY	Australia	**W87**	Shakah95	Egypt
**W6**	BARI-Gom-30	Bangladesh	**W47**	EGA KIDMAN	Australia	**W88**	Giza171	Egypt
**W7**	BARI-Gom-31	Bangladesh	**W48**	EGA WEDGETAIL	Australia	**W89**	JM22	China
**W8**	BARI-Gom-32	Bangladesh	**W49**	FANG	Australia	**W90**	YM20	China
**W9**	BAW-1147	Bangladesh	**W50**	Federation	Australia	**W91**	H-115	China
**W10**	BAW-1194	Bangladesh	**W51**	FRAME	Australia	**W92**	H-179	China
**W11**	BAW-1202	Bangladesh	**W52**	GAMENYA	Australia	**W93**	H-215	China
**W12**	BAW-1135	Bangladesh	**W53**	GILES	Australia	**W94**	Kzyl-Sark	Australia
**W13**	BAW-1293	Bangladesh	**W54**	GLADIUS	Australia	**W95**	ZM33	China
**W14**	BAW-1157	Bangladesh	**W55**	H45	Australia	**W96**	BANKS	Australia
**W15**	BAW-1208	Bangladesh	**W56**	HALBERD	Australia	**W97**	Mengavi	Australia
**W16**	BWSN-11	Bangladesh	**W57**	HOFED	Australia	**W98**	BATAVIA	Australia
**W17**	BWSN-13	Bangladesh	**W58**	KELALAC	Australia	**W99**	PYT-19	Bangladesh
**W18**	BWSN-14	Bangladesh	**W59**	KENNEDY	Australia	**W100**	BARI-Gom-29	Bangladesh
**W19**	BWSN-16	Bangladesh	**W60**	LIVINGSTON	Australia	**W101**	CM72 (check genotype)	USA
**W20**	BWSN-7	Bangladesh	**W61**	MACHETE	Australia			
**W21**	BWSN-22	Bangladesh	**W62**	MERINDA	Australia			
**W22**	BWSN-31	Bangladesh	**W63**	QAL2000	Australia			
**W23**	BWSN-33	Bangladesh	**W64**	ROWAN	Australia			
**W24**	BWSN-36	Bangladesh	**W65**	SHIELD	Australia			
**W25**	BWSN-38	Bangladesh	**W66**	STRZELECKI	Australia			
**W26**	BWSN-40	Bangladesh	**W67**	SUNBRI	Australia			
**W27**	BWSN-42	Bangladesh	**W68**	SUNBROOK	Australia			
**W28**	PYT-6	Bangladesh	**W69**	SUNCO	Australia			
**W29**	PYT-11	Bangladesh	**W70**	SUNFIELD	Australia			
**W30**	BARI-Gom-8	Bangladesh	**W71**	SUNSOFT 98	Australia			
**W31**	BWA-1	Bangladesh	**W72**	SUNSTATE	Australia			
**W32**	PYT-34	Bangladesh	**W73**	SUNVALE	Australia			
**W33**	AYT-10	Bangladesh	**W74**	SUNVEX	Australia			
**W34**	KRL-19	Bangladesh	**W75**	SUNZELL	Australia			
**W35**	AGT KATANA	Australia	**W76**	VULCAN	Australia			
**W36**	ANNUELLO	Australia	**W77**	WALLUP	Australia			
**W37**	BAXTER	Australia	**W78**	WESTONIA	Australia			
**W38**	BOLAC	Australia	**W79**	WYALKATCHEM	Australia			
**W39**	CARINYA	Australia	**W80**	YITPI	Australia			
**W40**	CASCADES	Australia	**W81**	Chinese spring	China			
**W41**	CORACK	Australia	**W82**	Suntop	Australia			
